# Substituting sedentary time with physical activity in youngest-old to oldest-old community-dwelling older adults: Associations with body composition

**DOI:** 10.3389/fpubh.2022.837213

**Published:** 2022-11-29

**Authors:** Ting-Fu Lai, Yung Liao, Ming-Chun Hsueh, Hsin-Yen Yen, Jong-Hwan Park, Jae Hyeok Chang

**Affiliations:** ^1^Department of Health Promotion and Health Education, National Taiwan Normal University, Taipei, Taiwan; ^2^Graduate Institute of Sport, Leisure and Hospitality Management, National Taiwan Normal University, Taipei, Taiwan; ^3^Master's Program of Transition and Leisure Education for Individuals With Disabilities, University of Taipei, Taipei, Taiwan; ^4^School of Gerontology Health Management, College of Nursing, Taipei Medical University, Taipei, Taiwan; ^5^Health Convergence Medicine Laboratory, Biomedical Research Institute, Pusan National University Hospital, Busan, South Korea; ^6^Department of Rehabilitation Medicine, School of Medicine, Pusan National University, Busan, South Korea

**Keywords:** adiposity, elderly, accelerometry, body composition, inactivity

## Abstract

**Introduction:**

Several studies have suggested that physical activity and sedentary behavior are strongly and independently associated with body composition and obesity. However, few studies have investigated whether substituting sedentary time with moderate-to-vigorous physical activity is associated with body composition in older adults, especially among those older than 75 years.

**Methods:**

This study examined the associations between replacing sedentary time with physical activity and obesity indices in a sample of 199 community-dwelling older Taiwanese adults (52.3% women; 80.6 ± 7.0 years). Physical activity and sedentary behavior were measured using the triaxial accelerometer (GT3X+, ActiGraph). Body composition indices were computed through a bioelectrical impedance analysis of body fat percentage and appendicular skeletal muscle mass index. Waist circumference and body mass index were measured by trained personnel. Isotemporal substitution analyses estimated these associations after adjusting for sociodemographic characteristics and nutritional status.

**Results:**

The study showed that substituting 30 min of sedentary behavior per day with moderate-to-vigorous physical activity was associated with lower body fat percentage (B = −1.408, 95% CI = −2.55, −0.264), body mass index (B = −0.681, 95% CI = −1.300, −0.061), and waist circumference (B = −2.301, 95% CI = −4.062, −0.539) after adjusting for covariates. Substituting 30 min of light physical activity per day with moderate-to-vigorous physical activity was associated with lower waist circumference (B = −2.230, 95% CI = −4.173, −0.287) after adjusting for covariates. Stratified analyses indicated that associations were stronger in youngest-old older adults, and in older adults with a normal nutritional status (vs. underweight status).

**Discussion:**

These findings confirm the importance of reducing sedentary behavior and increasing moderate-to-vigorous physical activity among older adults to improve their physical health, as well as highlighting the importance of taking into account nutritional status and age group.

## Introduction

As the aging population trends are higher globally, the number of older people is estimated to increase from 1 billion in 2020 to 2.1 billion by 2050 (1). Chronic ailments such as cardiovascular diseases and type two diabetes are increasingly prevalent in older individuals worldwide (2, 3). Studies have indicated that maintaining a healthy body weight in old age plays a vital role in preventing chronic diseases (4, 5) and that the prevalence of obesity has increased in high-income (6) as well as low to middle-income countries (7). Moreover, a longitudinal study indicated that long-term exposure to obesity is associated with poor performance of physical function in later life (8). Given that several studies have suggested that physical activity and sedentary behavior (SB) are strongly and independently associated with cardiac biomarkers and body composition (9–11), it is important to develop effective strategies for older populations.

Good nutritional status, physical activity, and SB play crucial roles in preventing metabolic syndrome and sarcopenia in older adults (12). Even in older adults aged 75 years and above, physical activity seems to delay age-related physiological decline, *via* its influence on several pathways, including improved cardiovascular fitness, decelerated sarcopenia, reduced adiposity, and improved immunity (13). Previous studies on obesity risk factors in older adults often highlighted the direct health benefits of light physical activity (LPA) and moderate-to-vigorous physical activity (MVPA) (14–16). According to the latest WHO guidelines on physical activity and SB, older adults should limit the amount of time spent being sedentary; replacing sedentary time with physical activity of any intensity provides health benefits (17).

Isotemporal substitution models have frequently been used in statistical analysis to explore the association between reallocating the time spent in SB and other health outcomes such as mortality, mental health, adiposity, fitness, and cardiometabolic biomarkers (18). These models assume that available time for physical activity and SB is limited—increasing the time spent on one activity, such as sedentary time, means less time for another activity, such as MVPA (19). This indicates that these activities are co-dependent. Previous studies have implied that substituting sedentary time for MVPA time had a beneficial effect on body mass index (BMI) (20), waist circumference (WC) (16, 21), and body fat percentage (15, 20). However, few studies have examined the associations between substituting SB with MVPA and body composition (including appendicular skeletal muscle) in older adults (18, 22). Moreover, previous systematic reviews revealed that most studies were with youngest-old adults (aged 65–74 years), and none were with middle (75–84 years) (21) or oldest-old adults (85 years and above) (23). Therefore, the present study aims to examine the association between body composition and replacing sedentary time with physical activity in youngest-old to oldest-old community-dwelling older adults. The outcomes were body composition [appendicular skeletal muscle mass index (ASMI), body fat percentage, etc.], BMI, and WC. Compared to the muscle mass, the ASMI is calculated as appendicular skeletal mass index (ASM)/height^2^ (ASM/m^2^) which correlates with nutrition and physical status and may be a more effective indicator than the muscle mass in preventing the sarcopenia (24). The hypothesis of this study is that replacing SB with physical activity of any intensity is associated with lower body fat percentage BMI, WC, and greater ASMI in all older adults.

## Materials and methods

This study uses a cross-sectional design and it was approved by the Research Ethics Committee of the National Taiwan University Hospital (REC Number: 202008046RINC).

### Participants

The study recruited 199 community-dwelling individuals. Inclusion criteria were individuals aged from 65 to 98 years (the proportion: youngest-old adults: 24.1%; middle-old adults: 44.7%; oldest-old adults: 31.2%), living in Taipei City—Taiwan, the ability to walk independently, no severe hearing or visual impairments, and consistently wearing a triaxial accelerometer for 7 days. The participants were recruited through two channels. The first was patients who had consulted a doctor at the National Taiwan University Hospital for medical issues. The other channel was patients who saw a doctor in the outpatient clinic of the same hospital. Participants were recruited through phone calls for verbal agreement. The sampling was carried out from August 2020 to March 2021. Participants from both channels provided informed consent.

### Obesity indices

#### Body mass index and waist circumference

BMI is calculated by dividing an individual's body weight in kilograms by the square of the person's height in meters. WC is measured at the middle point between the vertebral rib and the top of the ilium, roughly in line with the umbilicus (25).

#### Appendicular skeletal muscle mass index and body fat percentage

Body fat percentage (%) and ASMI (muscle mass/m^2^) were calculated using bioelectrical impedance analysis (“Babybot” — Netown, Taiwan) (26). ASMI is the sum of the muscle mass of a person's four limbs divided by the square of their height in meters (27). ASMI is used as an indicator of sarcopenia (defined as age-related loss of muscle mass and function) (28, 29). These anthropometric indices were measured by trained personnel.

### Objectively monitored physical activity and sedentary behavior

Participants were requested to wear a hip-mounted triaxial accelerometer (ActiGraph GT3X+, Pensacola, FL, USA) continuously for 7 days (30) to estimate the time spent in SB (≤ 99 counts/min), LPA (100–2019 counts/min), and MVPA (≥2020 counts/min). Instructions on how to wear and remove the accelerometer before and after taking a shower or performing water-based activities were given to participants by trained personnel. Sleep time was excluded using the sleep log. The vertical axis data from the accelerometers were then downloaded using the ActiLife software (version 6.0, Pensacola, FL, USA). Standard protocols for data collection and processing criteria of the triaxial accelerometer from ActiGraph were followed, as suggested by a systematic review (31). A valid day was 600 min or more of wear time; a zero-count was considered non-wear time, defined as no SB or physical activity for 60 consecutive minutes. Furthermore, data from at least four valid days (three weekdays and one weekend day) were classified as valid and included in the analysis.

### Covariates

Sociodemographic characteristics such as age, gender, educational level, and living status were self-reported. Participants were also asked to report their cigarette and alcohol use habits. They were also asked to report whether they were ever diagnosed with hypertension or diabetes. Because previous study inplies that with mini nutritional assessment score is low in elderly subjects with sarcopenia (32) and negatively associated with central obesity (33), nutritional status was assessed through mini nutritional assessment short-form questions (34). The participants were then categorized as either “normal nutrition” (12–14 points) or “at risk of malnutrition” (≤ 11 points) status.

### Statistical analyses

Descriptive analyses were conducted using the IBM SPSS 23.0 software (SPSS Inc., IBM, Chicago, IL, USA). Outcomes can be either continuous or dichotomous for isotemporal substitution modeling (19). Multiple linear regression models were used to analyze the theoretical effect of replacing 30 min of sedentary time with LPA and MVPA (35). The models considered all the LPA and MVPA activity variables simultaneously, with the exception of sedentary time. The total wear time variables and covariates were kept constant. Including the total wear-time variable made time isotemporal. The regression estimate for each activity variable in the model—unstandardized coefficients (B) with 95% confidence interval (CI)—demonstrated the effect of substituting 30 min of sedentary time with an equivalent LPA and MVPA time. All analyses were conducted using the SPSS 23.0 software for Windows (SPSS Inc., IBM, Chicago, IL, USA). Stratified analyses for nutritional status (normal vs. at risk of malnutrition) and age group (65–74 vs. 75 years old and above) were performed and are reported in the Appendix.

## Results

### Participant characteristics

All 199 individuals (52.3% women; 80.6 ± 7.0 years) aged 65 years and older (more than three quarters of them were older than or equal to 75 years old) were included in the analyses (Table 1). Most of the participants lived with others (90.5%) and had tertiary education (50.3%). The majority of them did not smoke cigarettes (93.0%) or drink alcohol (89.4%), were diagnosed with hypertension (58.8%), and did not have diabetes (66.3%). The participants' average BMI (24.3 ± 3.6 kg/m^2^) was on the cusp of obesity. The average duration for LPA, MVPA, SB, and wear time were 244.1 ± 86.2, 16.2 ± 24.6, 609.2 ± 79.0, and 869.5 ± 80.3 min/day (average days worn: 6.3 ± 0.8), respectively. In terms of obesity indices, participants' average WC (cm), body fat percentage (%), and ASMI (muscle mass/m^2^) were 85.8 ± 10.9, 29.5 ± 8.2, 7.7 ± 1.4, respectively.

**Table 1:** Characteristics of participants (*n* = 199).

**Variables**	**Mean ±SD**	**Categories**	***n***	**%**
Age (years)	80.6 ± 7.0	65–74 years	48	24.1%
		75+ years	151	75.9%
BMI	24.3 ± 3.6			
Sex		Women	104	48.94%
		Men	95	51.06%
Living status		Living with others	180	90.5%
		Living alone	19	9.5%
Educational level		Lower than university	99	49.7%
		University	100	50.3%
Smoking		No	185	93.0%
		Yes	14	7.0%
Drinking		No	178	89.4%
		Yes	21	10.6%
Hypertension		No	82	41.2%
		Yes	117	58.8%
Diabetes		No	132	66.3%
		Yes	67	33.7%
Nutritional status	10.1 ± 1.2	Normal	158	79.4%
		At risk of malnutrition	41	20.6%
MVPA (min/day)	16.2 ± 24.6			
LPA (min/day)	244.1 ± 86.1			
SB (min/day)	609.2 ± 79.0			
Wear time (min/day)	869.5 ± 80.3			
Number of days of wearing	6.3 ± 0.8			
Waist circumference (cm)	85.8± 10.9			
Body fat percentage (%)	29.5 ± 8.2			
ASMI (muscle mass/m^2^)	7.7 ± 1.4			
^*^Having risk of Sarcopenia		No	187	94.0
		Yes	12	6.0

* Cutoffs for ASMI are 7.05 kg/m^2^ for men and 5.85 kg/m^2^ for women which are practicability for screening sarcopenia.

### Isotemporal substitution model

The results of replacing 30 min of SB with LPA and MVPA on the risk factors of obesity using unadjusted and adjusted isotemporal substitution models are shown in Tables 2, 3. In the unadjusted model, the test results indicated that reallocating 30 min of SB per day with MVPA (increasing MVPA at the expense of SB) was associated with lower body fat percentage (*B* = −2.216, 95% CI = −3.695, −0.736). Replacing 30 min of LPA per day with MVPA was also associated with lower body fat percentage (*B* = −2.024, 95% = −3.669, −0.379). In the adjusted model, the test results indicated that reallocating 30 min of SB per day with MVPA (increasing MVPA at the expense of SB) was associated with lower body fat percentage (*B* = −1.408, 95% CI = −2.552, −0.264), BMI (*B* = −0.681, 95% CI = −1.300, −0.061), and WC (*B* = −2.301, 95% CI = −4.062, −0.539). Furthermore, reallocating 30 min of LPA per day with MVPA was also associated with lower WC (*B* = −2.230, 95% CI = −4.173, −0.287). There was no significant relationship between ASMI and reallocating any type of physical activity with SB. In stratified analysis of the adjusted model, the results indicated that reallocating 30 min of SB per day with MVPA was also associated with lower body fat percentage (*B* = −1.960, 95% CI = −3.387, −0.532), BMI (*B* = −0.766, 95% CI = −1.495, −0.036), and WC (*B* = −2.322, 95% CI = −4.150, −0.493) in the normal nutritional status group. Moreover, replacing 30 min of LPA per day with MVPA was associated with lower WC in this group. There were no significant associations between any indicators of body composition and reallocating any type of physical activity with SB in having risk of malnutrition group. In terms of stratified analysis of the adjusted model for age agroup, the results showed that replacing 30 min of SB per day with MVPA was associated with lower body fat percentage (*B* = −2.105, 95% CI = −4.177, −0.033) in the age group of 65–74 years. There were no significant associations between any indicators of body composition and replacing any type of physical activity with SB in the age group of 75 years old and above, middle-old (75–84 years), and oldest-old (85 years and above). Addiitonally, the null associations were also apparent when further excluding those who were with underweight nutritional status in two age group.

**Table 2:** Unadjusted isotemporal substitution models examining the associations of replacing 30 min sedentary behavior, LPA, and MVPA on body composition (*n* = 199).

**Analysis method**	**SB**			**LPA**			**MVPA**		
	**B**	**95% CI**	***p***	**B**	**95% CI**	***p***	**B**	**95% CI**	***p***
**ASMI (kg/m** ^**2**^ **)**
Replace SB with activity	Dropped			0.047	(−0.136, 0.042)	0.301	0.023	(−0.234, 0.281)	0.859
Replace LPA with activity	0.047	(−0.042, 0.136)	0.301	Dropped			0.070	(−0.216, 0.357)	0.630
Replace MVPA with activity	−0.023	(−0.281, 0.234)	0.859	0.070	(−0.357, 0.216)	0.630	Dropped		
**Body fat percentage (%)**
Replace SB with activity	Dropped			−0.191	(−0.702, 0.320)	0.461	–**2.216**	**(**–**3.695**, –**0.736)**	0.004*
Replace LPA with activity	0.191	(−0.320, 0.702)	0.461	Dropped			–**2.024**	**(**–**3.669**, –**0.379)**	0.016*
Replace MVPA with activity	**2.216**	**(0.736, 3.695)**	0.004*	**2.024**	**(0.379, 3.669)**	0.016*	Dropped		
**BMI (kg/m** ^**2**^ **)**
Replace SB with activity	Dropped			−0.049	(−0.270, 0.173)	0.666	−0.582	(−1.223, 0.059)	0.075
Replace LPA with activity	0.049	(−0.173, 0.270)	0.666	Dropped			−0.534	(−1.247, 0.180)	0.142
Replace MVPA with activity	0.582	(−0.059, 1.223)	0.075	0.534	(−0.180, 1.247)	0.142	Dropped		
**Waist circumference (cm)**
Replace SB with activity	Dropped			0.180	(−0.839, 0.478)	0.589	−1.848	(−3.753, 0.057)	0.057
Replace LPA with activity	0.180	(−0.478, 0.839)	0.589	Dropped			−1.667	(−3.786, 0.452)	0.122
Replace MVPA with activity	1.848	(−0.452, 3.786)	0.057	1.667	(−0.452, 3.786)	0.122	Dropped		

Bold values represent significant association between independent variables and outcome variables.

**Table 3:** Adjusted substitution models examining the associations of replacing 30 min sedentary behavior, LPA and MVPA on body composition (*n* = 199).

**Analysis method**	**SB**			**LPA**			**MVPA**		
	**B**	**95% CI**	***p***	**B**	**95% CI**	***p***	**B**	**95% CI**	***p***
**ASMI (kg/m** ^**2**^ **)**
Replace SB with activity	Dropped			−0.001	(−0.061, 0.059)	0.969	−0.121	(−0.297, 0.055)	0.176
Replace LPA with activity	0.001	(−0.059, 0.061)	0.969	Dropped			−0.120	(−0.314, 0.074)	0.225
Replace MVPA with activity	0.121	(−0.055, 0.297)	0.176	0.120	(−0.074, 0.314)	0.255	Dropped		
**Body fat percentage (%)**
Replace SB with activity	Dropped			−0.178	(−0.566, 0.210)	0.367	–**1.408**	**(**–**2.552**, –**0.264)**	0.016*
Replace LPA with activity	0.178	(0.210, 0.566)	0.367	Dropped			−1.230	(−2.492, 0.032)	0.056
Replace MVPA with activity	**1.408**	**(0.264, 2.552)**	0.016*	1.230	(−0.032, 2.49)	0.056	Dropped		
**BMI (kg/m** ^**2**^ **)**
Replace SB with activity	Dropped			−0.010	(−0.221, 0.200)	0.922	–**0.681**	**(**–**1.300**, –**0.061)**	0.032*
Replace LPA with activity	0.010	(−0.200, 0.221)	0.922	Dropped			−0.670	(−1.354, 0.014)	0.055
Replace MVPA with activity	**0.681**	(0.061, 1.300)	0.032*	0.670	(−0.014, 1.354)	0.055	Dropped		
**Waist circumference (cm)**
Replace SB with activity	Dropped			−0.070	(−0.668, 0.527)	0.816	–**2.301**	**(**–**4.062**, –**0.539)**	0.011*
Replace LPA with activity	0.070	(−0.527, 0.668)	0.816	Dropped			–**2.230**	**(**–**4.173**, –**0.287)**	0.025*
Replace MVPA with activity	**2.301**	**(4.062, 0.539)**	0.011*	2.230*	**(0.287, 4.173)**	0.025*	Dropped		

Adjusted for sociodemographics (age, sex, education, and living status) and health status (hypertension, diabetes, alcohol, smoking, and nutritional status) and monitor wear time; *p < 0.05.

Bold values represent significant association between independent variables and outcome variables.

## Discussion

Among factors related to obesity, physical activity has been recognized as a critical lifestyle factor to prevent or delay muscle loss and cardiovascular diseases with aging. Higher physical intensity helps maintain the skeletal muscle's aerobic capacity in older adults (36), reduces insulin resistance, and has an anti-inflammatory effect on the immune system (24). This study further presents a novel opportunity to investigate the effect of replacing SB with physical activity on the risks of obesity in community-dwelling older adults. The present study found that replacing 30 min of SB with the same amount of MVPA time is associated with better body fat percentage, BMI, and WC in a sample of community-dwelling older adults ranging in age from 65 to 98 years. Associations held when taking age, sex, sociodemographic characteristics, health status, and nutritional status into account. Moreover, a significant relationship was found between WC and replacing a period of LPA with MVPA. Therefore, MVPA is an essential daily activity associated with the risks of obesity. A 30-min reallocation of SB time to MVPA time was associated with ~1.41% lower body fat percentage, 0.68 kg/m^2^ lower BMI, and 2.30 cm lower WC. A 30-min reallocation of LPA time to MVPA time was associated with ~2.23 cm lower WC.

Previous studies on adults aged 65–80 have highlighted the benefits of replacing sedentary time with MVPA time on improved BMI, WC (15, 21), type two diabetes, body fat percentage (15), and muscle mass (23). Similarly, this study further finds that substituting 30 min of SB time for MVPA time is significantly associated with improved body fat percentage, BMI, and WC in a sample of youngest-old to oldest older adults. However, it is important to note that the results did not reach statistical significance in the adjusted model when examining associations in the age group 75 years and above (*n* = 151), suggesting that effects were stronger in the younger age group (65–74 years, *n* = 48). The null associations were also apparent when splitting the 75+ age group into middle-old (75–84 years) and oldest-old (85 years and above) and further excluding those who were with underweight nutritional status in two age group. Given that a previous prospective study also indicated that the associations between diet and physical activity and body composition may be weaker in mid to old-older adults because body weight, body fat, and muscle mass are generally lower in aging process (37), these findings warrant further investigation in a larger sample and with a more comprehensive assessment of health lifestyle.

Our results revealed that there were no statistical significance in replacing LPA with SB on obesity indices, while previous studies show that reallocating 30 min from sedentary time to LPA was associated with significant decreases in BMI (15, 21, 38), WC (15, 21), and body fat (38) in older adults. A potential explanation for this finding may be that the average BMI (mean: 24.3 kg/m^2^) and WC (mean: 85.8 cm) of our sample were lower than those studies (mean BMI: 28.1 kg/m^2^ and above; mean WC: 107 cm and above). A previous study also indicated that the magnitude of associations between PA and body composition is greater for higher intensity activities (33). As the sample size of this study was relatively small, it is possible that it wasn't sufficiently powered to detect effects of LPA. Our finding showed no effect on the muscle mass. Previous researches have investigated the above effect on the muscle mass. One cross-sectional study indicated that reallocating 1 h of MVPA time per day instead of SB time was associated with increased muscle mass (23). Another two studies showed that reallocating SB time to LPA time does not affect muscle mass (i.e., fat-free mass index) (38, 39). A possible reason could also be that the average age of our participants is older [80.1 vs. 64.6 (38) and 69.3 years (39)]. Nutrition such as dietary protein (40) and physical activity should be simultaneously considered (41) when maintaining or improving muscle mass. In terms of stratified analysis, the results indicated that the benefits of substituting SB with MVPA were apparent for normal nutritional status older adults. Although there is no study has investigated the association between replacing SB with PA on body composition in older adults at risk of malnutrition, one study implied that protein supplementation plus exercise training is effective in improving lean mass and muscle strength in frail older individuals which emphasized that nutrient intake and physical activity are both important to low weight older adults (32).

The study shows that MVPA is often the primary factor driving the relationship between daily activities and adiposity or obesity. However, reallocating SB time with LPA time is not associated with obesity risks because LPA intensity is not significant enough to reduce body fat or increase muscle mass. This finding is consistent with that of a previous study (23). This study suggests that replacing 30 min of SB with MVPA may be key to preventing obesity in older adults. This study can assist health professionals in designing appropriate strategies to delay age-related physiological decline and reduce the negative influences of an inactive lifestyle on the aging process.

### Strengths and limitations

To our knowledge, this is the first study to examine the effect of replacing SB time with LPA or MVPA time on appendicular skeletal muscle mass index by using isotemporal substitution analysis in youngest-old to oldest-old older adults. Moreover, we also measured the nutritional status of older adults as a covariate in the statistical model with the mini nutritional assessment. Because previous literature also indicated that with mini nutritional assessment score is low in elderly subjects with sarcopenia (42) and negatively associated with central obesity (32). Nevertheless, this study has certain limitations. First, the study's cross-sectional design requires interpreting the interchanging results as emphasizing the differences between groups with different activity patterns. Second, the study's sample size is relatively small and uses convenience sampling, and therefore, it cannot represent the Taiwanese population as a whole. Future studies should use a long-term study design and representative sample of older adults in the area. Third, the objective physical activity measurement (triaxial accelerometers, Actigraph GT3X+) cannot detect whether older adults are swimming, sitting, standing, lying down, or taking an afternoon nap. Thus, the time spent in SB, LPA, or MVPA can be over or underestimated. Finally, energy intake was not included in the study. For more comprehensive interventions in older adults' health, other indicators of health status (e.g., well-being and quality of life) is suggested to be included in future studies.

## Conclusion

In conclusion, this study has shown that replacing 30 min of SB with MVPA can reduce body fat percentage, BMI, and WC of community-dwelling older adults. The findings also highlight the importance of taking into account different stages of older adulthood as well as nutritional factors when intervening to reduce SB and increase physical activity.

## Data availability statement

The raw data supporting the conclusions of this article will be made available by the authors, without undue reservation.

## Ethics statement

The study was approved by the Research Ethics Committee of National Taiwan University Hospital (REC Number: 202008046RINC). The patients/participants provided their written informed consent to participate in this study. Written informed consent was obtained from the individual(s) for the publication of any potentially identifiable images or data included in this article.

## Author contributions

T-FL: formal analysis and writing—original draft. YL: conceptualization, data curation, and writing—review and editing. M-CH: data curation and writing—review and editing. H-YY, J-HP, and JC: writing—review and editing. All authors contributed to the article and approved the submitted version.

## Funding

This work was supported by Institute of Information and Communications Technology Planning and Evaluation (IITP) grant funded by the Korea Government (MSIT) (No. 2022-0-00501, Development of Wearable Device Assessment Technology).

## Conflict of interest

The authors declare that the research was conducted in the absence of any commercial or financial relationships that could be construed as a potential conflict of interest.

## Publisher's note

All claims expressed in this article are solely those of the authors and do not necessarily represent those of their affiliated organizations, or those of the publisher, the editors and the reviewers. Any product that may be evaluated in this article, or claim that may be made by its manufacturer, is not guaranteed or endorsed by the publisher.

## Abbreviations

SB: sedentary behavior
MVPA: moderate-to-vigorous physical activity
LPA: light physical activity
ASMI: appendicular skeletal mass
WC: waist circumference.
