# Supplementary material for: Substituting sedentary time with physical activity in youngest-old to oldest-old community-dwelling older adults: Associations with body composition
Source: Front Public Health. 2022 Nov 29;10:837213. doi: 10.3389/fpubh.2022.837213 (PMC9746713; doi:10.3389/fpubh.2022.837213)
Supplement: Supplementary file 1 [file Table_1.docx]

Appendix 1.1.1 Unadjusted Isotemporal Substitution Models Examining the Associations of Replacing 30 min Sedentary Behavior, LPA and MVPA on risk factors of obesity in normal nutritional status group (n= 158)

| Analysis Method | SB | | | LPA | | | | MVPA | | |
| --- | --- | --- | --- | --- | --- | --- | --- | --- | --- | --- |
|  | B | 95%CI | *p* | B | 95%CI | | *p* | B | 95%CI | *p* |
| ASMI (kg/m^2^) |  |  |  |  |  | |  |  |  |  |
| Replace SB with activity | Dropped |  |  | -0.045 | (-0.157, 0.067) | | 0.429 | -0.021 | (-0.299, 0.256) | 0.879 |
| Replace LPA with activity | 0.045 | (-0.067, 0.157) | 0.429 | Dropped |  | |  | 0.024 | (-0.291, 0.338) | 0.882 |
| Replace MVPA with activity | 0.021 | (-0.256, 0.299) | 0.879 | -0.024 | (-0.338, 0.291) | 0.882 | Dropped |  |  |  |
| Body fat percentage (%) |  |  |  |  |  | |  |  |  |  |
| Replace SB with activity | Dropped |  |  | -0.058 | (-0.682, 0.566) | | 0.854 | -2.266 | (-3.807, -0.725) | 0.004* |
| Replace LPA with activity | 0.058 | (-0.566, 0.682) | 0.854 | Dropped |  | |  | -2.208 | (-3.957, -0.458) | 0.014* |
| Replace MVPA with activity | 2.266 | (0.725, 3.807,) | 0.004* | 2.208 | (0.458, 3.957) | | 0.014* | Dropped |  |  |
| BMI (kg/m^2^) |  |  |  |  |  | |  |  |  |  |
| Replace SB with activity | Dropped |  |  | -0.024 | (-0.291, 0.242) | | 0.857 | -0.680 | (-1.338, -0.021) | 0.043* |
| Replace LPA with activity | 0.024 | (-0.242, 0.291) | 0.857 | Dropped |  | |  | -0.103 | (-0.334, 0.138) | 0.085 |
| Replace MVPA with activity | 0.680 | (1.338, 0.021) | 0.043* | 0.103 | (-0.138, 0.334) | | 0.085 | Dropped |  |  |
| Waist circumference (cm) |  |  |  |  |  | |  |  |  |  |
| Replace SB with activity | Dropped |  |  | -0.189 | (-0.968, 0.590) | | 0.633 | -2.038 | (-3.962, -0.113) | 0.038* |
| Replace LPA with activity | 0.189 | (-0.590, 0.968) | 0.633 | Dropped |  | |  | -1.849 | (-4.034, 0.337) | 0.097 |
| Replace MVPA with activity | 2.038 | (0.113, 3.962) | 0.038* | 1.849 | (-0.337, 4.034) | | 0.097 | Dropped |  |  |

Appendix 1.1.2 Adjusted Isotemporal Substitution Models Examining the Associations of Replacing 30 min Sedentary Behavior, LPA and MVPA on risk factors of obesity in normal nutritional status group (n= 158)

| Analysis Method | SB | | | LPA | | | MVPA | | |
| --- | --- | --- | --- | --- | --- | --- | --- | --- | --- |
|  | B | 95%CI | *p* | B | 95%CI | *p* | B | 95%CI | *p* |
| ASMI (kg/m^2^) |  |  |  |  |  |  |  |  |  |
| Replace SB with activity | Dropped |  |  | 0.021 | (-0.052, 0.095) | 0.570 | -0.147 | (-0.331, 0.037) | 0.117 |
| Replace LPA with activity | -0.021 | (-0.095, 0.052) | 0.570 | Dropped |  |  | -0.168 | (-0.373, 0.037) | 0.107 |
| Replace MVPA with activity | 0.147 | (-0.037, 0.331) | 0.117 | 0.168 | (-0.037, 0.373) | 0.107 | Dropped |  |  |
| Body fat percentage (%) |  |  |  |  |  |  |  |  |  |
| Replace SB with activity | Dropped |  |  | -0.300 | (-0.869, 0.268) | 0.298 | -1.960 | (-3.387, -0.532) | 0.007* |
| Replace LPA with activity | 0.300 | (-0.268, 0.869) | 0.298 | Dropped |  |  | -1.659 | (-3.248, -0.070) | 0.041* |
| Replace MVPA with activity | 1.960 | (0.532, 3.387) | 0.007* | 1.659 | (0.070, 3.248) | 0.041* | Dropped |  |  |
| BMI (kg/m^2^) |  |  |  |  |  |  |  |  |  |
| Replace SB with activity | Dropped |  |  | -0.021 | (-0.282, 0.241) | 0.876 | -0.766 | (-1.495, -0.036) | 0.040* |
| Replace LPA with activity | 0.021 | (-0.241, 0.282) | 0.876 | Dropped |  |  | -0.670 | (-1.354, 0.014) | 0.055 |
| Replace MVPA with activity | 0.786 | (0.131, 1.442) | 0.019* | 0.766 | (0.036, 1.495) | 0.040* | Dropped |  |  |
| Waist circumference (cm) |  |  |  |  |  |  |  |  |  |
| Replace SB with activity | Dropped |  |  | -0.079 | (-0.808, 0.650) | 0.831 | -2.322 | (-4.150, -0.493) | 0.013* |
| Replace LPA with activity | 0.079 | (-0.650, 0.808) | 0.831 | Dropped |  |  | -2.243 | (-4.278, -0.207) | 0.031* |
| Replace MVPA with activity | 2.322 | (0.493, 4.150) | 0.013* | 2.243 | (4.278, 0.207) | 0.031* | Dropped |  |  |

Adjusted for sociodemographics (age, sex, education, living status) and health status (hypertension, diabetes, alcohol, smoking, and monitor wear time; * p <0.05.

Appendix 1.2.1 Unadjusted Isotemporal Substitution Models Examining the Associations of Replacing 30 min Sedentary Behavior, LPA and MVPA on risk factors of obesity in having risk of malnutrition group (n= 41)

| Analysis Method | SB | | | LPA | | | MVPA | | |
| --- | --- | --- | --- | --- | --- | --- | --- | --- | --- |
|  | B | 95%CI | *p* | B | 95%CI | *p* | B | 95%CI | *p* |
| ASMI (kg/m^2^) |  |  |  |  |  |  |  |  |  |
| Replace SB with activity | Dropped |  |  | -0.019 | (-0.151, 0.114) | 0.778 | 0.175 | (-0.685, 1.034) | 0.683 |
| Replace LPA with activity | 0.019 | (-0.114, 0.151) | 0.778 | Dropped |  |  | 0.193 | (-0.725, 1.112) | 0.673 |
| Replace MVPA with activity | -0.175 | (-1.034, 0.685) | 0.683 | -0.193 | (-1.112, 0.725) | 0.673 | Dropped |  |  |
| Body fat percentage (%) |  |  |  |  |  |  |  |  |  |
| Replace SB with activity | Dropped |  |  | -0.175 | (-1.124, 0.773) | 0.710 | -4.583 | (-10.733, 1.568) | 0.140 |
| Replace LPA with activity | 0.175 | (-0.773, 1.124) | 0.710 | Dropped |  |  | -4.407 | (-10.983, 2.168) | 0.183 |
| Replace MVPA with activity | 4.583 | (-1.568, 10.733) | 0.140 | 4.407 | (-2.168, 10.983) | 0.183 | Dropped |  |  |
| BMI (kg/m^2^) |  |  |  |  |  |  |  |  |  |
| Replace SB with activity | Dropped |  |  | 0.046 | (-0.339, 0.431) | 0.809 | -1.119 | (-3.615, 1.376) | 0.369 |
| Replace LPA with activity | -0.046 | (-0.431, 0.339) | 0.809 | Dropped |  |  | -1.165 | (-3.833, 1.502) | 0.382 |
| Replace MVPA with activity | 1.119 | (-1.376, 3.615,) | 0.369 | 0.373 | (-3.024, 3.770) | 0.824 | Dropped |  |  |
| Waist circumference (cm) |  |  |  |  |  |  |  |  |  |
| Replace SB with activity | Dropped |  |  | 0.376 | (-0.877, 1.629) | 0.547 | -4.823 | (-12.949, 3.303) | 0.237 |
| Replace LPA with activity | -0.376 | (-1.629, 0.877) | 0.547 | Dropped |  |  | -5.199 | (-13.886, 3.487) | 0.233 |
| Replace MVPA with activity | 4.823 | (-3.303, 12.949) | 0.237 | 5.199 | (-3.487, 13.886) | 0.233 | Dropped |  |  |

Appendix 1.2.2 Adjusted Isotemporal Substitution Models Examining the Associations of Replacing 30 min Sedentary Behavior, LPA and MVPA on risk factors of obesity in having risk of malnutrition group (n= 41)

| Analysis Method | SB | | | LPA | | | MVPA | | |
| --- | --- | --- | --- | --- | --- | --- | --- | --- | --- |
|  | B | 95%CI | *p* | B | 95%CI | *p* | B | 95%CI | *p* |
| ASMI (kg/m^2^) |  |  |  |  |  |  |  |  |  |
| Replace SB with activity | Dropped |  |  | -0.011 | (-0.121, 0.099) | 0.841 | 0.192 | (-0.587, 0.972) | 0.618 |
| Replace LPA with activity | 0.011 | (-0.099, 0.121) | 0.841 | Dropped |  |  | 0.203 | (-0.628, 1.035) | 0.621 |
| Replace MVPA with activity | -0.192 | (-0.972, 0.587) | 0.618 | -0.203 | (-1.035, 0.628) | 0.621 | Dropped |  |  |
| Body fat percentage (%) |  |  |  |  |  |  |  |  |  |
| Replace SB with activity | Dropped |  |  | -0.282 | (-1.311, 0.748) | 0.580 | -2.943 | (-10.245, 4.360) | 0.417 |
| Replace LPA with activity | 0.282 | (-0.748, 1.311) | 0.580 | Dropped |  |  | -2.661 | (-10.448, 5.127) | 0.490 |
| Replace MVPA with activity | -2.943 | (-4.360, 10.245) | 0.417 | 2.661 | (-5.127, 10.448) | 0.490 | Dropped |  |  |
| BMI (kg/m^2^) |  |  |  |  |  |  |  |  |  |
| Replace SB with activity | Dropped |  |  | 0.030 | (-0.419, 0.479) | 0.893 | -0.343 | (-3.529, 2.842) | 0.827 |
| Replace LPA with activity | 0.030 | (-0.479, 0.419) | 0.893 | Dropped |  |  | -0.373 | (-3.770, 3.024) | 0.824 |
| Replace MVPA with activity | 0.343 | (-2.842, 3.529) | 0.827 | 0.373 | (-3.024, 3.770) | 0.824 | Dropped |  |  |
| Waist circumference (cm) |  |  |  |  |  |  |  |  |  |
| Replace SB with activity | Dropped |  |  | 0.226 | (-1.111, 1.562) | 0.732 | -0.389 | (-9.867, 9.088) | 0.934 |
| Replace LPA with activity | -0.226 | (-1.562, 1.111) | 0.732 | Dropped |  |  | -0.615 | (-10.722, 9.491) | 0.902 |
| Replace MVPA with activity | 0.389 | (-9.088, 9.867) | 0.934 | 0.615 | (-9.491, 10.722) | 0.902 | Dropped |  |  |

Adjusted for sociodemographics (age, sex, education, living status) and health status (hypertension, diabetes, alcohol, smoking, and monitor wear time; * p <0.05.

Appendix 1.3.1 Unadjusted Isotemporal Substitution Models Examining the Associations of Replacing 30 min Sedentary Behavior, LPA and MVPA on body composition in age group of 65 to 74 years (n= 48)

| Analysis Method | SB | | | LPA | | | MVPA | | |
| --- | --- | --- | --- | --- | --- | --- | --- | --- | --- |
|  | B | 95%CI | *p* | B | 95%CI | *p* | B | 95%CI | *p* |
| ASMI (kg/m^2^) |  |  |  |  |  |  |  |  |  |
| Replace SB with activity | Dropped |  |  | -0.150 | (-0.331, 0.032) | 0.105 | 0.091 | (-0.479, 0.295) | 0.636 |
| Replace LPA with activity | 0.150 | (-0.032, 0.331) | 0.105 | Dropped |  |  | 0.058 | (-0.357, 0.473) | 0.779 |
| Replace MVPA with activity | -0.091 | (-0.295, 0.479) | 0.636 | -0.058 | (-0.473, 0.357) | 0.779 | Dropped |  |  |
| Body fat percentage (%) |  |  |  |  |  |  |  |  |  |
| Replace SB with activity | Dropped |  |  | 0.026 | (-0.890, 0.941,) | 0.955 | -2.071 | (-4.016, -0.127) | 0.037* |
| Replace LPA with activity | -0.026 | (-0.941, 0.890) | 0.955 | Dropped |  |  | -2.097 | (-4.185, -0.009) | 0.049 |
| Replace MVPA with activity | 2.071 | (0.127, 4.016) | 0.037* | 2.097 | (0.009, 4.185) | 0.049 | Dropped |  |  |
| BMI (kg/m^2^) |  |  |  |  |  |  |  |  |  |
| Replace SB with activity | Dropped |  |  | -0.123 | (-0.587, 0.341) | 0.596 | -0.691 | (-1.676, 0.293) | 0.164 |
| Replace LPA with activity | 0.123 | (-0.341, 0.587) | 0.596 | Dropped |  |  | -0.569 | (-1.626, 0.489) | 0.285 |
| Replace MVPA with activity | 0.691 | (-0.293, 1.676) | 0.164 | 0.569 | (-0.489, 1.626) | 0.285 | Dropped |  |  |
| Waist circumference (cm) |  |  |  |  |  |  |  |  |  |
| Replace SB with activity | Dropped |  |  | -0.544 | (-1.876, 0.788) | 0.415 | -2.000 | (-4.829, 0.829) | 0.161 |
| Replace LPA with activity | 0.544 | (-0.788, 1.876) | 0.415 | Dropped |  |  | -1.456 | (-4.494, 1.582) | 0.339 |
| Replace MVPA with activity | 2.000 | (-0.829, 4.829) | 0.161 | 1.456 | (-1.582, 4.494) | 0.339 | Dropped |  |  |

Appendix 1.3.2 Adjusted Isotemporal Substitution Models Examining the Associations of Replacing 30 min Sedentary Behavior, LPA and MVPA on body composition in age group of 65 to 74 years (n= 48)

| Analysis Method | SB | | | LPA | | | MVPA | | |
| --- | --- | --- | --- | --- | --- | --- | --- | --- | --- |
|  | B | 95%CI | *p* | B | 95%CI | *p* | B | 95%CI | *p* |
| ASMI (kg/m^2^) |  |  |  |  |  |  |  |  |  |
| Replace SB with activity | Dropped |  |  | -0.007 | (-0.145, 0.131) | 0.919 | -0.294 | (-0.594, 0.007) | 0.055 |
| Replace LPA with activity | 0.007 | (-0.131, 0.145) | 0.919 | Dropped |  |  | -0.287 | (-0.619, 0.046) | 0.089 |
| Replace MVPA with activity | 0.294 | (-0.007, 0.594) | 0.055 | 0.287 | (-0.046, 0.619) | 0.089 | Dropped |  |  |
| Body fat percentage (%) |  |  |  |  |  |  |  |  |  |
| Replace SB with activity | Dropped |  |  | -0.357 | (-1.311, 0.452) | 0.452 | -2.105 | (-4.177, -0.033) | 0.047* |
| Replace LPA with activity | 0.357 | (-0.452, 1.311) | 0.452 | Dropped |  |  | -1.747 | (-4.040, 0.545) | 0.131 |
| Replace MVPA with activity | 2.105 | (0.033, 4.177) | 0.047* | 1.747 | (-0.545, 4.040) | 0.131 | Dropped |  |  |
| BMI (kg/m^2^) |  |  |  |  |  |  |  |  |  |
| Replace SB with activity | Dropped |  |  | -0.038 | (-0.550, 0.475) | 0.882 | -0.975 | (-2.089, 0.138) | 0.084 |
| Replace LPA with activity | 0.038 | (-0.475, 0.550) | 0.882 | Dropped |  |  | -0.937 | (-2.169, 0.294) | 0.131 |
| Replace MVPA with activity | 0.975 | (-0.138, 2.089) | 0.084 | 0.937 | (-0.294, 2.169) | 0.131 | Dropped |  |  |
| Waist circumference (cm) |  |  |  |  |  |  |  |  |  |
| Replace SB with activity | Dropped |  |  | -0.249 | (-1.710, 1.211) | 0.731 | -2.625 | (-5.799, 0.548) | 0.102 |
| Replace LPA with activity | 0.249 | (-1.211, 1.710) | 0.731 | Dropped |  |  | -2.376 | (-5.887, 1.135) | 0.186 |
| Replace MVPA with activity | 2.625 | (-0.548, 5.799) | 0.102 | 2.376 | (-1.135, 5.887) | 0.186 | Dropped |  |  |

Adjusted for sociodemographics (age, sex, education, living status) and health status (hypertension, diabetes, alcohol, smoking, and nutritional status) and monitor wear time; * p <0.05

Appendix 1.4.1 Unadjusted Isotemporal Substitution Models Examining the Associations of Replacing 30 min Sedentary Behavior, LPA and MVPA on body composition in age group of 75 years old and above (n= 151)

| Analysis Method | SB | | | LPA | | | MVPA | | |
| --- | --- | --- | --- | --- | --- | --- | --- | --- | --- |
|  | B | 95%CI | *p* | B | 95%CI | *p* | B | 95%CI | *p* |
| ASMI (kg/m^2^) |  |  |  |  |  |  |  |  |  |
| Replace SB with activity | Dropped |  |  | -0.019 | (-0.130, 0.093,) | 0.740 | 0.046 | (-0.393, 0.485) | 0.836 |
| Replace LPA with activity | 0.019 | (-0.093, 0.130) | 0.740 | Dropped |  |  | 0.065 | (-0.427, 0.557) | 0.795 |
| Replace MVPA with activity | -0.046 | (-0.485, 0.393) | 0.836 | -0.065 | (-0.557, 0.427) | 0.795 | Dropped |  |  |
| Body fat percentage (%) |  |  |  |  |  |  |  |  |  |
| Replace SB with activity | Dropped |  |  | -0.365 | (-1.024, 0.294) | 0.276 | -2.842 | (-5.437, -0.248) | 0.032* |
| Replace LPA with activity | 0.365 | (-0.294, 1.024) | 0.276 | Dropped |  |  | -2.478 | (-5.386, 0.431) | 0.094 |
| Replace MVPA with activity | 2.842 | (0.248, 5.437) | 0.032* | 2.478 | (-0.431, 5.386) | 0.094 | Dropped |  |  |
| BMI (kg/m^2^) |  |  |  |  |  |  |  |  |  |
| Replace SB with activity | Dropped |  |  | -0.069 | (-0.333, 0.196) | 0.608 | -0.801 | (-1.840, 0.239) | 0.130 |
| Replace LPA with activity | 0.069 | (-0.196, 0.333) | 0.608 | Dropped |  |  | -0.732 | (-1.898, 0.434) | 0.217 |
| Replace MVPA with activity | 0.801 | (-0.239, 1.840) | 0.130 | 0.732 | (-0.434, 1.898) | 0.217 | Dropped |  |  |
| Waist circumference (cm) |  |  |  |  |  |  |  |  |  |
| Replace SB with activity | Dropped |  |  | -0.083 | (-0.893, 0.727) | 0.839 | -2.907 | (-6.094, 0.280) | 0.074 |
| Replace LPA with activity | 0.083 | (-0.727, 0.893) | 0.839 | Dropped |  |  | -2.824 | (-6.397, 0.749) | 0.120 |
| Replace MVPA with activity | 2.907 | (-0.280, 6.094) | 0.074 | 2.824 | (-0.749, 6.397) | 0.120 | Dropped |  |  |

Appendix 1.4.2 Adjusted Isotemporal Substitution Models Examining the Associations of Replacing 30 min Sedentary Behavior, LPA and MVPA on body composition in age group of 75 years old and above (n= 151)

| Analysis Method | SB | | | LPA | | | MVPA | | |
| --- | --- | --- | --- | --- | --- | --- | --- | --- | --- |
|  | B | 95%CI | *p* | B | 95%CI | *p* | B | 95%CI | *p* |
| ASMI (kg/m^2^) |  |  |  |  |  |  |  |  |  |
| Replace SB with activity | Dropped |  |  | -0.011 | (-0.083, 0.061) | 0.766 | 0.003 | (-0.289, 0.295) | 0.986 |
| Replace LPA with activity | 0.011 | (-0.061, 0.083) | 0.764 | Dropped |  |  | 0.014 | (-0.310, 0.337) | 0.934 |
| Replace MVPA with activity | -0.003 | (-0.295, 0.289) | 0.986 | -0.014 | (-0.337, 0.310) | 0.934 | Dropped |  |  |
| Body fat percentage (%) |  |  |  |  |  |  |  |  |  |
| Replace SB with activity | Dropped |  |  | -0.232 | (-0.827, 0.363) | 0.441 | -2.102 | (-4.501, 0.298) | 0.086 |
| Replace LPA with activity | 0.232 | (-0.363, 0.827) | 0.441 | Dropped |  |  | -1.869 | (-4.529, 0.791) | 0.167 |
| Replace MVPA with activity | 2.102 | (-0.298, 4.501) | 0.086 | 1.869 | (-0.791, 4.529) | 0.167 | Dropped |  |  |
| BMI (kg/m^2^) |  |  |  |  |  |  |  |  |  |
| Replace SB with activity | Dropped |  |  | -0.017 | (-0.272, 0.237) | 0.894 | -0.628 | (-1.654, 0.398) | 0.228 |
| Replace LPA with activity | 0.017 | (-0.237, 0.272) | 0.894 | Dropped |  |  | -0.611 | (-1.748, 0.527) | 0.290 |
| Replace MVPA with activity | 0.628 | (-0.398, 1.654) | 0.228 | 0.609 | (-0.524, 1.742) | 0.290 | Dropped |  |  |
| Waist circumference (cm) |  |  |  |  |  |  |  |  |  |
| Replace SB with activity | Dropped |  |  | 0.030 | (-0.710, 0.769) | 0.937 | -2.423 | (-5.405, 0.559) | 0.110 |
| Replace LPA with activity | -0.030 | (-0.769, 0.710) | 0.937 | Dropped |  |  | -2.453 | (-5.758, 0.852) | 0.145 |
| Replace MVPA with activity | 2.423 | (-0.559, 5.405) | 0.110 | 2.453 | (-0.852, 5.758) | 0.145 | Dropped |  |  |

Adjusted for sociodemographics (age, sex, education, living status) and health status (hypertension, diabetes, alcohol, smoking, and nutritional status) and monitor wear time; * p <0.05
